# The “Lab4treat” Outreach Experience: Preparation of Sustainable Magnetic Nanomaterials for Remediation of Model Wastewater

**DOI:** 10.3390/molecules26113361

**Published:** 2021-06-02

**Authors:** Maria Laura Tummino, Roberto Nisticò, Flavia Franzoso, Alessandra Bianco Prevot, Paola Calza, Enzo Laurenti, Maria Cristina Paganini, Dominique Scalarone, Giuliana Magnacca

**Affiliations:** 1 Department of Chemistry, University of Torino, Via P. Giuria 7, 10125 Torino, Italy; marialaura.tummino@unito.it (M.L.T.); flavia.franzoso@gmail.com (F.F.); alessandra.biancoprevot@unito.it (A.B.P.); paola.calza@unito.it (P.C.); enzo.laurenti@unito.it (E.L.); mariacristina.paganini@unito.it (M.C.P.); dominique.scalarone@unito.it (D.S.); 2 Department of Applied Science and Technology DISAT, Polytechnic of Torino, C.so Duca degli Abruzzi 24, 10129 Torino, Italy; 3 NIS Interdepartmental Centre, University of Torino, Via P. Giuria 7, 10125 Torino, Italy

**Keywords:** water pollution, magnetic material, magnetic recovery, adsorption, biomasses valorization, chemical education, hands-on learning

## Abstract

The Lab4treat experience has been developed to demonstrate the use of magnetic materials in environmental applications. It was projected in the frame of the European project Mat4Treat, and it was tested several times in front of different audiences ranging from school students to the general public in training and/or divulgation events. The experience lends itself to discuss several aspects of actuality, physics and chemistry, which can be explained by modulating the discussion depth level, in order to meet the interests of younger or more experienced people and expand their knowledge. The topic is relevant, dealing with the recycling of urban waste and water depollution. The paper is placed within the field of water treatment for contaminant removal; therefore, a rich collection of recent (and less recent) papers dealing with magnetic materials and environmental issues is described in the Introduction section. In addition, the paper contains a detailed description of the experiment and a list of the possible topics which can be developed during the activity. The experimental approach makes the comprehension of scientific phenomena effective, and, from this perspective, the paper can be considered to be an example of interactive teaching.

## 1. Introduction

The literature offers a considerable amount of papers and reviews reporting iron-based magnetic materials for environmental applications, mostly related to adsorption and catalytic activity. The success of such compounds derives from the possibility to achieve easy recovery after their use and from their definition as “ecomaterials”, due to biological safety, reusability, recyclability and chemical stability [1].

The importance of the topic can be evidenced by a study of the literature. A study carried out on the Web of Science platform using the keywords “magnetic materials” AND “environmental applications” in this century reports the first paper published in 2006 about the use of Fe_3_O_4_ in the removal of As [2], which has received 342 citations up to the present. Thereafter, the number of papers concerning the use of magnetic materials applied to environmental applications for polluted water and soil treatments increased significantly, and since 2009 many other papers have been published, several of them included in a 2010 review [3] collecting studies of water purification using magnetic assistance. Limiting the selection to papers cited at least 50 times, we can mainly find works on magnetite (Fe_3_O_4_) or maghemite (γ-Fe_2_O_3_) used for the magnetic recovery of adsorbing active phases. In several cases, the active phases showed a certain affinity towards polar contaminants or metals [4,5,6,7,8,9,10,11,12,13,14], but also the adsorption of apolar substrates was investigated [15,16,17]. In other publications, less common magnetic materials were studied: for instance, Co [18] or FeNi_3_ [19]. Although the function of iron containing materials was principally determined by their magnetic features, iron oxides were successfully applied in photocatalytic processes, as widely described in a 2016 review [20] reporting on the synthesis and photocatalytic applications of iron oxide-based magnetic materials. More recently, similar aspects were revisited, and other aspects were investigated; Table 1 reports a selection of papers collected from recent literature of the period 2018–2021, which could give a clear idea of the multiple uses possible for magnetic materials in environmental applications.

Magnetic materials include: (i) the classical Fe_3_O_4_, easily formed by the coprecipitation reaction, but also prepared with tunable shapes and behaviors using sol-gel and hydrothermal pathways [24,31]; (ii) MnFe_2_O_3_, which is a good photocatalyst in activating H_2_O_2_ or peroxymonosulfate for substrate oxidation [25,35,36,39]; (iii) CoFe_2_O_4_, often employed as an adsorbent [33,41]; (iv) among others, the zero valent iron Fe^0^ (ZVI), typically produced by reduction from oxides, which covers a special role due its very high magnetization that makes it suitable in responding very efficiently to applied magnetic fields for a fast separation from aqueous media [1,30].

Among the possible environmental applications, water depollution is the main field where magnetic materials find very promising exploitation. Several pollutants can be removed by adsorption, but precipitation, reduction and oxidation are also important mechanisms. ZVI can be taken as an example, as it plays different interesting roles, as described in [1]; for instance, it holds a good potential to act as a reducing agent for contaminants under anaerobic conditions. During the redox reaction between ZVI and contaminants, Fe^2+^ is oxidized to Fe^3+^, which can form ferric hydroxide Fe(OH)_3_ as a very effective flocculant, facilitating the removal of various water pollutants. On the other hand, under aerobic conditions in the presence of dissolved oxygen, ZVI transfers two electrons to an oxygen molecule to produce hydrogen peroxide. The formed H_2_O_2_ and Fe^2+^ represent Fenton’s reagents, which can generate strong oxidizing agents as hydroxyl radicals, capable of oxidizing a wide range of organic pollutants.

However, as mentioned above, the main application of iron-based systems still remains in the field of adsorption. Iron-based nanoparticles, in fact, can be functionalized very easily to tune their removal efficiency towards several contaminants ranging from organic molecules to metals and ions [1,21,22,23,24,25,26,27,28,29,30,31,32,33,34,40,41].

Usually, magnetic particles are used as core–shell composite materials together with silica, clay composites, carbonaceous materials, polymers and wastes, which induce good interaction with several substrates and guarantee a good stability of the magnetic domains against the dissolution in acidic media or oxidation [26]. Among the different types of functionalizing agents, biomaterials are actually widely investigated, as they conjugate good or excellent performance with environmental compatibility and low costs. For instance, alginate-based composites feature biocompatibility and cost efficiency with no toxicity. Talbot et al. [42] reported the synthesis of alginate/maghemite nanoparticles for the adsorption of methylene blue; on the other hand, Mohammadi et al. [43] fabricated superparamagnetic sodium alginate-coated Fe_3_O_4_ nanoparticles, well performing in the adsorption of malachite green.

Great attention has been also focused on waste-based materials, representing a sustainable method for waste valorization. The most common materials exploit agricultural residues, but waste from the paper industry and sludge from water treatment plants have also been studied. Safarik and coworkers synthetized magnetic adsorbents by putting a suspension of magnetite in contact with powdered peanut husk and pine sawdust, respectively [44,45]. Zuorro et al. [46] prepared a coffee silverskin-derived magnetic material that was tested for methylene blue adsorption. Stan et al. [47] studied four different magnetite–starch materials for the removal of optilan blue dye obtaining efficiencies between 72% and 89%. Jodeh et al. [48] prepared magnetic cellulose-based materials from olive industry solid waste and tested the adsorption performance of these materials with methylene blue. Sriplai et al. [22] reviewed the possibility to use residual carbon sources to feed bacteria producing bacterial cellulose to be functionalized with magnetic nanoparticles synthesized in the cellulose pores.

In this wide scenario, the present paper describes the preparation and application of core–shell magnetite nanoparticles, employing soluble organic matter extracted from urban “green” residues, tested as sustainable adsorbent materials for aqueous contaminant removal [49,50,51].

The production (and management) of huge amounts of waste, byproducts of our consumerist society, requests strategies for their smart reuse. In this context, new concepts proposed by the green economy promote the progressive reduction of both (bio)waste volumes and its hazardousness, favoring the proliferation of novel alternative processes capable of satisfying social, environmental, and economic needs, and guaranteeing adequate protection of natural resources.

Among the different classes of waste, biodegradable organic waste represents a quantitatively relevant category with a remarkable intrinsic problem due to its putrescible nature. The term “biodegradable organic waste” refers to biowaste derived from parks and gardens, food waste from kitchen and restaurants and the food and beverage industry. On the basis of recent research, the total amount of biodegradable organic waste produced by the EU member states was estimated as being ca. 76.5–102 Mt per year for the food and garden waste category (including the municipal undifferentiated waste fraction) [52], whereas the food and beverage industry waste fraction was estimated as being ca. 37 Mt per year [53]. Currently, municipal organic biowaste is hard to be directly exploited and/or recycled due to its compositional heterogeneity together with the high content of water. On the one hand, these last aspects remarkably affect their management and disposal. On the other hand, the concentration of biowaste within confined (urban) areas makes this biomass an appealing sustainable resource that deserves proper treatment for its potential exploitation. Among the different biologic processes developed for the treatment of biomasses, aerobic digestion (i.e., composting) is the most commonly adopted biological treatment. Briefly, biomass composting allows for obtaining soil improvers and fertilizers salable to the horticulture market.

This aspect is not the only added value of composted biowaste, as the humic substances (which are the major organic component in natural soils), also formed by biowaste during the composting process, possess other valuable behaviors, which are at the base of the research developed at the University of Torino (Department of Chemistry) in the framework of the Biochemenergy [54], EnvironBOS [55] and Mat4treat projects [56]. Such humic-like substances, named biobased substances (BBS in the following) are lignin-derived supramolecular aggregates with a very complex chemical structure and can be extracted by acid/basic treatment from the composted matrix [57]. Beyond the organic portion, BBS contain a non-negligible amount of inorganic species (salts or oxides deriving from the original waste or from the extraction procedure). Moreover, BBS have amphiphilic properties due to the copresence of both hydrophilic and hydrophobic moieties, making them exploitable as detergents and auxiliaries for the textiles industry [58,59]. In the last years, particular emphasis has been devoted to the application of such biowaste-derived products in the removal of wastewater pollutants. In particular, the use of BBS in the preparation of magnetic hybrid materials to be exploited in the heterogeneous remediation process of contaminated water is the most recent frontier that has been object of investigation by our research team [60].

All these themes, generally confined within a debate among experts, need to be communicated to the public in order to share knowledge and, consequently, create confidence towards science and scientific/technologic research. This is one of the aspects concerning the concept of Open Science, which is becoming very relevant for European policy. In this framework, an outreach activity developed about the use of a magnetic material for depollution purposes has been projected and presented in several events of public engagement by our research team. Depending on the audience, the phenomena occurring during the experiment can be described modulating the explanation of the target knowledge: the integration of the practical approach facilitates the comprehension of the conceptual contents. As already stated, the main goal of such experience is aimed at raising the citizens’ awareness toward the binomial environment pollution and physical/chemical remediation, creating a good feeling toward science, technology, engineering and mathematics (STEM) disciplines. For this purpose, it is rather challenging to maintain a rigorous scientific language and conceptualization while engaging different audiences. Indeed, public engagement must not be conceived as a tool to attract young people by merely simplifying complex topics, but rather as a powerful opportunity to bring young people closer to science by contributing to the progressive construction of correct knowledge that allows them to exercise the role of an active, aware and critical citizen. Given these premises, the “Lab4treat” laboratory was thought to make new generations of citizens more involved in environmental concerns and possible solutions.

The practical aim of this didactic experience is the production of magnetic materials containing BBS acting as adsorbents for the removal of a dye (taken as reference pollutant) from water. The experience does not comprise the procedure to extract BBS from compost (which is a long and relatively complex process); thus, a commercially available humic acid was used.

In a typical laboratory session of ca. 2 h, the Lab4treat experience is organized as follows: 30 min devoted to the preliminary discussion on renewable sources of raw materials (evidencing the importance of the differential collection of organic residues for recycle and reuse), 10 min devoted to the presentation of the experiment, 40 min for the preparation of the magnetic material, 20 min for the water depollution procedure (i.e., removal of a dye from water) and the final 20 min dedicated to the final discussion. In general, for a valuable experience, the practical sessions should be organized by dividing the audience into groups of three to four people.

## 2. Materials and Methods

Magnetic iron oxides were prepared by using anhydrous ferric chloride FeCl_3_ 6H_2_O (CAS 7705-08-0, purity > 98%, Sigma-Aldrich, St. Louis, Missouri, United States) and ferrous sulphate heptahydrate FeSO_4_ 7H_2_O (CAS 7782-63-0, purity > 99.5%, Sigma-Aldrich). Other reagents used were: ammonium hydroxide solution (CAS 1336-21-6, NH_3_ essay 28–30%, Merck, Darmstadt, Germany) and crystal violet (CV, C_25_H_30_N_3_Cl, CAS 548-62-9, purity > 90%, Merck). To avoid the extraction procedure of humic substances from compost, simplifying the operations, commercial humic acids (CAS 1415-93-6, purity > 80%, Merck) can be employed. All solutions can be prepared with commercially available deionized water. All chemicals were used without further purification. For magnetic separation, a common neodymium magnet (diameter 0.8 cm, thickness 0.3 cm), usually sold in hardware shops, was used.

## 3. Results: The Experiment

### 3.1. Magnetic Material Preparation

**Figure 1:**
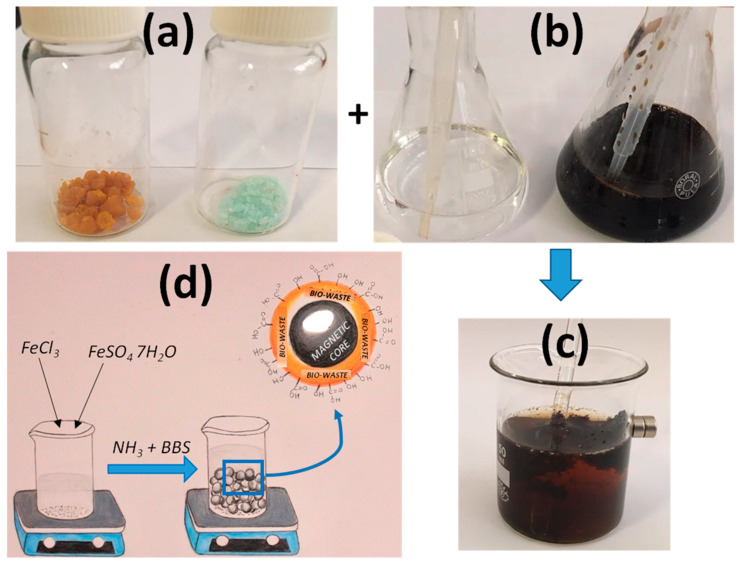
Steps for the preparation of magnetic material. Powders of FeCl_3_ and FeSO_4_ (**a**) are dissolved in water and combined with NH_3_ and BBS solution (**b**) under a fume hood or in open air; after stirring and ripening the magnetic material is formed (**c**); (**d**) reports a pictorial representation of the synthesis.

Magnetic hybrid nanomaterials are prepared following a well-consolidated procedure from the literature [61]. Briefly, 1 g of humic acid is dissolved into 100 mL of water. Since the dissolution can take some time, it can be shortened by increasing the pH of the solution by the addition of few drops of NH_3_ 30%. Then, 1.5 g of FeCl_3_ ∙ 6 H_2_O and 1.0 g FeSO_4_ ∙ 7 H_2_O are dissolved in a 100-mL container (a beaker or a plastic glass) containing 25 mL of water, obtaining an orange and clear solution. Afterwards, 2.5 mL of 28–30% NH_3_ (the use of a plastic pipette is suggested) and 12.5 mL of the previously prepared humic acid solution are added, in fast sequence, to the solution containing the iron salts. The orange solution becomes instantaneously black and dense because the magnetic oxide precipitates in the basic environment. Since NH_3_ produces some irritating vapors, it is, therefore, suggested to work under a fume hood or, in its absence, in open air or near an open window. The solid needs to ripen, thus it is left resting for 5 min. After this time, the black solid deposits at the bottom of the container in presence of a separated pale yellow liquid. When a magnet is brought close to the container side (always outside the container), after the ripening, the black solid is attracted by the magnet and the magnet sticks at the container side thanks to the interaction with the magnetic particles (Figure 1).

Part of the suspension can be transferred into a plastic Petri dish (or a simple plastic dish) using the pipette (three of four full load pipettes are sufficient). We recommend taking the solid at the bottom of the container, as we need to separate it from the yellow liquid. The magnet placed at the bottom of the dish (always outside the container) attracts the solid and allows the removal of the liquid (using a pipette or simply allowing it to flow out of the container while keeping the solid blocked with the magnet). The magnet is removed at the end of this phase.

### 3.2. Water Depollution Experiment

**Figure 2:**
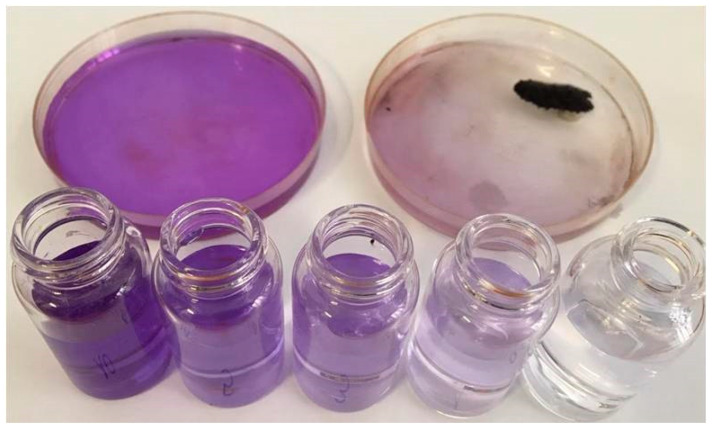
Use of the magnetic material for removing CV from water. Upper images: dye solution before (left) and after (right) the contact with BBS-functionalized magnetic material and recovery of BBS-functionalized magnetic material with a magnet. Bottom section: CV solutions with decreasing molar concentrations (from left to right 10.0, 5.0, 3.0, 1.0, 0.5 M, respectively) to be used as optical comparisons to determine the residual concentration of the dye after the adsorption process. In this picture, the CV concentration before and after the adsorption varies from 10 ppm to about 1–2 ppm.

The magnetic nanomaterial is now ready for the adsorption experiment and 4–5 mL of crystal violet (CV) aqueous solution in a concentration of 10 mg L^−1^ can be added to the dish. The solution is dark violet when added. The dish is gently shaken with a rotary movement in order to favor the contact between the magnetic material and the dye. Afterwards, the magnet can be placed again next to the dish bottom (always outside the container): it attracts the magnetic solid and allows for observing the lighter color of the solution after CV removal (Figure 2).

The next paragraph is dedicated to the exploration of different physical-chemical topics that can be taken into account during a scientific divulgation event. The slant of the discussion in those situations must be modulated to be comprehensible to a general public, but here, an in-depth discussion will be provided, also creating a sort of compendium about several aspects concerning magnetic materials for water depollution that are generally taken for granted in research articles.

## 4. Discussion: Chemical and Physical Phenomena Occurring during the Experiment: Analytical, Inorganic, Physical Chemistry for Dummies

### 4.1. Collection of Organic Waste, Aerobic and Anaerobic Digestion, Production of Gas and Compost. BBS Extraction and Composition

In order to obtain the BBS to be used for the preparation of core–shell magnetic material, starting from organic waste, it is necessary to go through several steps.

First of all, the different types of chemicals present in organic waste should undergo chemical transformation, “leveling” their composition until the “humification” of the waste is attained (that is, the transformation of most organic molecules into humic-like substances). Indeed, organic waste derived from park and garden trimming is mostly characterized by cellulosic and lignocellulosic structure, whereas organic waste coming from the food chain also contains fats, proteins and other carbohydrates. At the present time, there are two main routes to treat this waste in order to recover useful products: aerobic and anaerobic digestion.

*Aerobic digestion* (commonly named “composting”) is a treatment more suitable for lignocellulosic materials, such as “green” waste from parks and gardens. It consists of a two-step aerobic biological degradation: (1) High rate. Different microorganisms, in the presence of atmospheric oxygen, can oxidize the most degradable organic fraction (i.e., sugars, acids, amino acids) to CO_2_ and water with release of heat, reaching a temperature higher than 70 °C, suitable to destroy eventual pathogens. A partially transformed organic residue is obtained. (2) Curing phase. In slightly less oxidative conditions, specific microorganisms synthesize complex tridimensional organic polymers, very similar to humic substances naturally present in soils. The final product is named compost and can be used as a soil improver both in gardening and agriculture. Moreover, in mixture with peat, compost can improve the quality of artificial soils.

*Anaerobic digestion* occurs in the absence of oxygen, when part of the organic waste is transformed by anaerobic microorganisms to biogas, namely a mixture of methane (44–61%), CO_2_ (25–50%) and, to a minor extent, aqueous vapor, H_2_S, H_2_ and other gases. This biogas can be used as biofuel, contributing to decrease in the emission of greenhouse gases. The production of biogas is the result of a series of reactions such as hydrolysis (fats to fat acids, carbohydrates to sugars, proteins to amino acids), acetogenesis (production of acetic acid) and finally the reduction to methane (methanogenesis).

The lignin fraction is the principal component of the solid residue obtained at the end of the anaerobic digestion, and it can be sent for its further transformation to aerobic treatment. Therefore, the integration of aerobic and anaerobic treatment allows the complete transformation of organic waste into compost and biogas.

ACEA Pinerolese Industriale carries out these treatments on collected urban wastes in the plant placed near Pinerolo (Torino, Italy) [62].

Substances featuring as humic acid substances, i.e., BBS, can be sourced from compost by exploiting their difference in solubility and dimension from other components. Compost can be dissolved in alkaline solution, then BBS can be isolated either by reacidification at pH < 4.5 followed by their precipitation and filtration, or by ultrafiltration and drying of the retentate; indeed, their higher dimension compared to other components yields to their retention by ultrafiltration membranes.

BBS are a rather complex mixture of macromolecules (weight in the range of 70–460 Kg mol^−1^), that can differ in carbon content as well as functional groups. In general, BBS structure contains long alkyl chain, with aromatic rings, and several other groups such as carboxylates, amino, phenolic and keto groups. All these groups contribute to the amphiphilic behavior of BBS, embedding hydrophilic and hydrophobic moieties [63].

### 4.2. Coprecipitation Synthesis for the Preparation of the Magnetic Material

Fe_3_O_4_ can be synthesized following several reaction pathways [20], but the easiest one is coprecipitation, since it allows for the preparation of a material with good magnetization properties and good dispersion. The coprecipitation method is widely used to prepare several materials. It starts by mixing anion and cation solutions (precursors, in our case, Fe^2+^, Fe^3+^ and OH^−^ groups) in a concentration high enough to cause the nucleation and growth of particles of the desired material (in our case, the magnetic phases magnetite Fe_3_O_4_ and maghemite γ-Fe_2_O_3_) which agglomerate and precipitate as a solid in the reaction medium. One of the most important steps in this synthesis is the Ostwald ripening, which is the process of the disappearance of small particles or droplets by dissolution and deposition on the larger particles or droplets. The driving force for Ostwald ripening is the difference in solubility between the small and the large particles. The smaller particles (with a higher radius of curvature) are more soluble than larger ones (with a lower radius of curvature). With time, the smaller particles or droplets dissolve, and their molecules diffuse in the bulk and become deposited on the larger ones. This results in a shift of the particle or droplet size distribution to larger values. In our experiments we use the iron salts that are polar species formed by cationic (positively charged) and anionic (negatively charged) species held together by electrostatic attraction. Due to this, salts are generally soluble in water since water molecules, which are polar, tend to hydrate the single ions, forming a surrounding shell around them and favoring their dissolution. Generally, the hydration enthalpy is higher than the lattice energy (the energy that keeps together the material), and this leads to a rapid dissolution of the salts. This phenomenon is not observed for all materials, since there are also poorly soluble compounds for which this dissolution process is limited to a certain concentration level. For instance, this is the case of some iron compounds, e.g., the iron hydroxides Fe(OH)_2_ and Fe(OH)_3_ and the oxides magnetite Fe_3_O_4_ and maghemite γ-Fe_2_O_3_, these latter ones being the subject of the Lab4treat experience. All these compounds easily precipitate (forming solid species) at a basic pH (i.e., in the presence of high concentration of OH^−^). When a particular limit of concentration (named solubility) is reached, the solute starts evolving in the formation of solid particles that cannot be further dissolved, and they collapse and form a layer of solid material at the bottom of the container due to the action of gravity.

### 4.3. Role of Humic Acid in the Preparation of Magnetic Material: Interaction of BBS Carboxylate Groups with Material Iron Cations. Protection Against Material Oxidation. Exposition of Reactive Moieties for Substrate Capture

Humic acids (or BBS) are limitedly soluble compounds, which are efficiently dissolved in water at weak basic pH, when the carboxylic functional groups present in their complex structure are dissociated in the form of –COO^−^ (negatively charged). As described before, the introduction of ammonia in the reaction environment containing Fe^2+^ and Fe^3+^ salts causes a fast increase in pH which favors the precipitation of the magnetic iron oxides Fe_3_O_4_ or γ-Fe_2_O_3_. The simultaneous addition of the basic solution containing BBS in the dissociated form induces the formation of electrostatic interactions between the carboxylate negative groups of BBS and the positively charged iron ions still present at the surface of the oxide particles. Such interactions lead to the formation of an organic coating/shell surrounding the magnetic particles [64]. This phenomenon is interesting for two reasons: (i) the organic coating preserves the magnetic iron oxide from the air-induced oxidation which might cause the conversion of the magnetic magnetite into the non-magnetic red hematite α-Fe_2_O_3_, which is the main reason for the loss of magnetic properties of these systems over time, and (ii) a magnetic hybrid inorganic-organic materials is obtained in a single step process. The water depollution experiments show very well how the magnetic core of the hybrid material is exploitable for facilitating the recovery of the material after its use, whereas the organic shell is useful as an active substrate for the sequestration of substances such as dyes, metal ions or other pollutants present in water (see [60] and references therein).

### 4.4. Introduction to Magnetism and Use in Environmental Applications

The Lab4treat experience is also a good opportunity to present and discuss magnetism and its related phenomena, as well as the integration of magnetic materials into ecofriendly clean-up processes. In detail, several forms of magnetism are available in nature, but the only ones that are macroscopically visible are basically two: ferromagnetism and ferrimagnetism [65]. Although all magnetic phenomena are related to the motions of electrons, ferromagnetism can be assumed as a strong magnetic phenomenon due to “electrons coupling” with the consequent formation of magnetic domains (e.g., this is the case of metallic powders). On the contrary, ferrimagnetism is a “structural phenomenon” occurring in materials organized into two interpenetrating structures with nonequal balance between the electrons’ magnetic moments (e.g., this is the case of magnetite and other oxide powders). However, when both classes of materials are organized into small nanostructures (of ca. 20 nm, assumed as “single domain”), they can change their direction of magnetization with time/temperature, and this phenomenon is named superparamagnetism. Hybrid particles prepared during the Lab4treat experience are superparamagnetic and can be exploited in wastewater treatments based on adsorption or photo-activated processes [64,65]. Both these processes have a critical issue, which is the recovery of the adsorbent/active species, which will be discussed in the next paragraph; nevertheless, as we will conclude after next paragraph, the possibility of exploiting the magnetic response of these materials to recover them from the purified liquid medium is a really interesting approach.

#### Industrial Applications of Magnetic Systems: Magnetic Separation

According to the literature, the downstream section is usually the most costly part of an industrial plant [66]. This is particularly true for a wastewater treatment plant, where the large variety of contaminant species (which need to be removed) deserves a very complex multistep approach. This manuscript already highlighted the possibility of using magnet-sensitive or magnetic-responsive (nano)materials as either sequestrating agents or photocatalysts in Advanced Oxidation Processes (AOPs) for water clean-up treatments. However, the effective recovery of these promising systems still deserves attention due to several engineering concerns (e.g., the method of applying the external magnetic field, as well as the magnetic separation of magnet-responsive nanoparticles from a dynamic flux of water) [64]. Among the different magnetic systems, superparamagnetic nanoparticles (e.g., magnetite) are able in the changing direction of magnetization with time/temperature fluctuations, thus preventing magnetic attraction between particles [67]. In this context, Sinha and coworkers [68] proposed an interesting study focused on the magnetophoretic separation of magnetic nanoparticles in a microfluidic channel under the influence of an external magnetic field applied, whereas, in a recent study, Leong et al. [69] classified magnetic separation into two distinct classes, namely high gradient magnetic separation (HGMS) and low gradient magnetic separation (LGMS). 

LGMS is the simplest setup, as the separation of magnetic materials from the liquid suspension is performed typically by a handheld permanent magnet that generates a nonhomogeneous magnetic field which favors nanoparticle migration towards the magnetic source [69]. On the contrary, HGMS has been designed as an interesting technological solution which allows for the integration of magnetic materials in industrial plants, thus turning magnetism as a real industrially exploitable alternative to more traditional approaches. As reported by Ge and co-workers [70], HGMS has been a successfully used in separating/filtrating weakly magnetic (nano)particles from fluid suspensions, guaranteeing high selectivity and efficiency, environmental friendliness and economic advantages. In detail, HGMS consists of applying a local magnetic field gradient using steel wire meshes. In the presence of a local magnetic field, magnetic particles can be strongly captured by the magnetized matrix when passing through it. Conversely, captured particles might be recovered by simply reducing the applied magnetic field to zero [70,71]. Lastly, Menzel et al. [72] reported the removal of magnetite from a mineral oil liquid suspension at a high amount.

### 4.5. Evaluation of Adsorption Capacities. Lambert–Beer law and Evaluation of Adsorbed Amounts

By analyzing the interaction between the solid material and the organic dye, it is possible to recognize an adsorption process in which the dye molecules dissolved in water are attracted by the solid material’s surface thanks to a reciprocal affinity. In fact, it must be noticed that both species (namely BBS and CV) are organic molecules with many polar and apolar functionalities, and consequently they are able to easily interact. In general, the sorption phenomena are related to molecules (at the liquid or gaseous phase) interacting and accumulating at a solid surface via either physical (physisorption) or chemical (chemisorption) interaction. The physical interactions are weak interaction forces generated by asymmetric interactions of the solid surface atoms, causing interactions with surrounding molecules. This phenomenon is typical for porous solids which have several voids within their structure and high values of surface area. On the contrary, the chemical interaction (or chemisorption) relies on energetically strong specific interaction with the formation of chemical bonds (covalent or electrostatic), such as in the case of BBS-functionalized magnetic material, as previously described.

A material can be classified as a good adsorbent on the basis of its sorption capacity, expressed as the amount of molecules accumulated at the surface before reaching the saturation point, and the time required for reaching this level (for real application it should be very short). In all cases, sorption processes are based on spontaneous equilibrium reactions, strongly influenced by the temperature, the nature of the adsorbent (solid) and the adsorbate (liquid/gaseous molecules), their relative amount and their reciprocal affinity. Every time the solid material presents a high surface area or a high affinity toward the adsorbate, the sorption process is strongly favored and, in some cases, can be considered as irreversible. However, adsorption is always an equilibrium process, and the opposite phenomenon (desorption) can also take place under certain conditions (such as a variation of temperature, or a proper washing step). This last process is fundamental for the industrial/practical application of the solid material, since it allows for the regeneration of its surface, increasing adsorbent recyclability.

To evaluate the extent of the adsorption, it is necessary to measure the amount of adsorbate captured by the adsorbent. The appropriate method of measure depends on the nature of the adsorptive; for instance, a reading of the pressure could be appropriate in the case of gases, whereas, dealing with a dye absorbing the VIS light, spectroscopic methods are typically applied. In the latter case, it is necessary to exploit the Lambert–Beer law (Equation (1)) where A: absorbance, ε: molar absorptivity coefficient, b: optical path length and C: concentration.

A = εbC(1)

**Figure 3:**
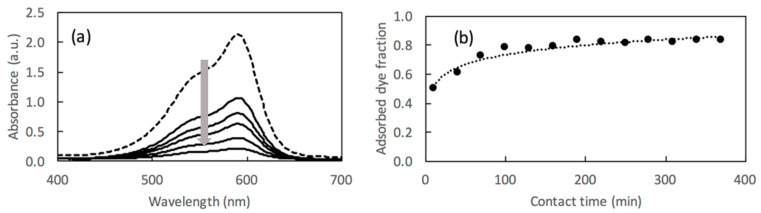
UV-vis spectra of CV solution before (broken-line curve) and after (solid-line curves) a prolonged contact with BBS-functionalized magnetic material for 6 h (**a**), and relative adsorbed dye fraction trend as determined by reading 592 nm absorbance (**b**). The grey arrow in section (**a**) indicates the trend of the absorption in time.

The Lambert–Beer law allows for converting the absorption intensities into concentrations considering a known value of b and a constant value of ε. In the case of CV, solution ε = 86500 M^−1^cm^−1^ at λ = 592 nm. The spectra recorded maintaining the dye in contact with adsorbing material for 6 h are reported in Figure 3a. The quantification of the adsorption, reported as adsorbed fraction, is shown in Figure 3b. When an instrument is not available for these evaluations, it is possible to compare the intensity of the solution color with a previously prepared optical scale, as the one reported in Figure 2, allowing for the comparison of the intensity of the color to the related concentration.

### 4.6. Perspective towards the Photocatalytic Abatement of the Captured Pollutants (BBS as Photosensitizers in Photocatalytic Processes)

A further perspective after and beyond the adsorption is the complete mineralization of the adsorbed pollutants, namely their transformation to simpler molecules such as CO_2_, H_2_O and inorganic salts [73]. For this purpose, a valid solution is represented by photocatalysis, in which a substance/material that can absorb light and promote the formation of reactive species (called photocatalyst or photosensitizer) is able to initiate a series of reactions disrupting the organic pollutants [60,74,75]. In this context, BBS can play a role as a photosensitizing species when subjected to solar light irradiation in the same way as the humic substances present in the natural environment work as photo-activated decontaminating agents. In the homogeneous phase, BBS (dissolved in water) have been proven to be efficient in the removal of naphtalensuphonates, azo-dyes and monochlorophenols, with good abatement results, as well as in terms of residual toxicity (the BBS themselves can be considered not toxic before and after irradiation) [60]. Heterogenized (supported) BBS, as the magnetic hybrids described above, have been also employed to photodegrade differently charged dyes [76], phenols [77,78], caffeine [37,61], carbamazepine [79], diclofenac [80] and other contaminants of emerging concern [81]. It is worth recalling that the heterogenization allows for the recovery and reuse of the active material, improving the sustainability of the whole process. Moreover, a peculiar situation that adopts BBS as photoactive substances, both in homogeneous and heterogeneous phase, is adding them in the presence of trivalent iron in order to induce the so-called photo-Fenton reaction [82]. In conclusion, the possibility to use BBS in various conditions and with different target molecules makes BBS ecofriendly and versatile tools for a synergistic action of sequestration and degradation of pollutants.

## 5. Conclusions

This experience can lead to the achievement of several goals, such as:

(1) acquiring manual skills by means of simple synthetic procedures, in particular evaluating the reactivity of chemicals after mixing reactants to obtain new substances;
(2) verifying the magnetic properties of the material synthesized by using a simple external commercially available magnet;
(3) verifying the capability of interactions between different materials, in particular focusing on the capacity of the produced material for “capturing” dye molecules, thus cleaning the aqueous solution.

Additionally, by providing adequate contextualization (as reported in the previous paragraphs), the Lab4treat experience can provide effective (hopefully) sensitizing action on a new generation of citizens toward the importance of environmental protection and waste management.

This paper can inspire other researchers to project scientific divulgation events and help them to easily find information about several phenomena concerning the preparation and application of magnetic materials for environmental remediation. 

## Figures and Tables

**Table 1:** Selection of literature papers and reviews concerning the use of magnetic materials in environmental applications (The table colors divide the materials on the basis of their action mechanism against target pollutants).

Ref.	First Author, Year	Type of Article	Materials	Action Mechanism	Targets	Notes
Materials for adsorption						
[21]	Wang 2020	Review	Magnetic porous organic frameworks	Adsorption	Emerging contaminants, metals, polycyclic aromatic hydrocarbons, other organic pollutants	
[22]	Sriplai 2021	Review	Magnetic bacterial cellulose	Adsorption	Heavy metal ions	Reuse of C-sources for bacterial metabolism
[23]	Imran Ali 2019	Research article	Biomagnetic membrane capsules	Adsorption	Malachite green	Encapsulation in polyvinyl alcohol/alginate
[24]	Nisar Ali 2019	Review	Fe-based magnetic compounds with high wettability properties	Adsorption and separation	Oil–water separation, dye-based pollutants, heavy metals	Overview of magnetic material synthesis, interfacial materials
[25]	Cui 2018	Research article	MnFe_2_O_4_-cellulose aerogel	Adsorption	Cu^2+^	
[26]	Peralta 2020	Review	Nanoabsorbents with magnetic core	Adsorption	Organic pollutants	
[27]	Han 2019	Research article	MOF-based magnetic materials	Adsorption	Congo red	
[28]	Gupta 2021	Research article	Fe_2_O_3_-activated bakelite nanocomposites	Adsorption	Victoria blue dye	
[29]	Medeiros 2019	Research article	Mesoporous composites glycerol-based with magnetic core	Adsorption	Organic contaminants (methylene blue, indigo carmine)	Reuse of glycerol by pyrolysis
[30]	Cui 2019	Research article	Fe/C	Adsorption	Cr(VI)	Pyrolysis of glycerol base precursors,
[31]	Liu 2020	Review	Fe_3_O_4_-based composites	Adsorption	Dye removal, oily wastewater treatment	Overview of Fe_3_O_4_ synthesis, pyrolysis and surface functionalization
[32]	Plohl 2021	Research article	Magnetic nanostructures with lysine	Adsorption	Heavy metals	
[33]	Matos Oliveira 2020	Research article	CoFe_2_O_4_ and natural organic matter	Adsorption	Polycyclic aromatic hydrocarbons, metals	
Materials with multiple actions						
[34]	Yi 2020	Review	Magnetic biochar	Adsorption, catalytic activation of H_2_O_2_ and persulfate	Adsorption of heavy metals, nuclear and organic pollutants, inorganic ions	Study of papers in the period 2011–2019
[1]	Singh 2019	Review	Zero valent iron, several materials	Precipitation, reduction, oxidation		
Materials for catalysis/photocatalysis						
[35]	Fu 2019	Research article	Biochar + MnFe_2_O_4_	Peroxymonosulfate activation	Organic pollutants	
[36]	He 2018	Research article	Reduced graphene oxide-MnFe_2_O_4_	Fenton-like, sunlight excitation	Malachite green	
[37]	Palma 2018	Research article	Magnetic Bio-based substances nanoparticles	(Photo)Fenton-like processes	Caffeine	
[38]	Tanaka 2019	Review	Iron-oxide-based nanoarchitectures, hybridization with inorganic and C-based materials	Catalysis	Air pollution, catalytic oxidation of CO	
[39]	Manohar 2021	Research article	MnFe_2_O_4_	Photocatalysis	Rhodamine B	
Promising materials to test						
[40]	Muños Medina 2020	Research article	Fe/FeO_x_/citrate	Adsorption	Good potential for environmental applications	High stability
[41]	Ravanello Mariosi 2020	Research article	La-doped spinel CoFe_2_O_4_	Adsorption	Good potential for environmental applications	

## Data Availability

Data is contained within the article.

## Sample Availability

Samples of the compounds are not available from the authors.
